# A Case of Psoriasis Concurrently Complicated by Sacroiliitis and Myalgic Encephalomyelitis/Chronic Fatigue Syndrome

**DOI:** 10.7759/cureus.92435

**Published:** 2025-09-16

**Authors:** Mami Iijima, Tomomitsu Miyagaki, Kaori Nakajima, Takafumi Kadono, Hidenori Watabe

**Affiliations:** 1 Dermatology, St. Marianna University School of Medicine, Kawasaki, JPN

**Keywords:** il-17 inhibitor, ixekizumab, leptin adiponectin, myalgic encephalomyelitis/chronic fatigue syndrome (cfs), "psoriasis, " "psoriatic arthritis, upadacitinib

## Abstract

Myalgic encephalomyelitis/chronic fatigue syndrome (ME/CFS) is a systemic chronic disorder characterized by persistent and unexplained severe fatigue. Recent population-based studies have revealed that patients with chronic inflammatory skin dermatoses, including psoriasis, are more likely to develop ME/CFS. Here, we report a case of psoriasis, whose exacerbation occurred concurrently with the development of sacroiliitis and the onset of ME/CFS. The pathogenesis of ME/CFS has not yet been fully elucidated, while inflammatory cytokines are involved in dysregulated interactions among the nervous, immune, and endocrine systems in the disease. We discussed the shared immunological abnormalities of psoriasis and ME/CFS based on previous literature. Our case contributes to the understanding of the association between psoriasis and ME/CFS.

## Introduction

Myalgic encephalomyelitis/chronic fatigue syndrome (ME/CFS) is a systemic chronic disorder showing persistent and unexplained severe fatigue as its primary symptom. Currently, the term ME/CFS is used internationally, often in conjunction with its former designation, CFS. Recent population-based studies have revealed that patients with chronic inflammatory skin dermatoses, such as atopic dermatitis and psoriasis, are more likely to develop ME/CFS [1,2]. A retrospective cohort study from Taiwan revealed that patients with psoriasis exhibited a significantly higher incidence of ME/CFS compared to non-psoriasis controls (hazard ratio (HR): 1.48), and the incidence of the syndrome in patients with psoriasis was 0.37 per 100 person-years [2]. Notably, although the risk in males was comparable to that in females in the general populations, the risk was greater among males in patients with psoriasis (HR: 2.05) [2]. In general populations, a higher age is a risk factor for the development of ME/CFS, and psoriasis patients aged ≥60 years are highly susceptible to ME/CFS compared to non-psoriasis controls (HR: 2.32) [2]. These findings suggest a potential pathophysiological link between psoriasis and ME/CFS, possibly mediated by shared mechanisms of immune dysregulation and systemic inflammation. However, the exact mechanisms remain unclear. In this report, we present a case of psoriasis in which the exacerbation of skin lesions and the development of sacroiliitis, which is the inflammation of joints connecting the sacrum to the ilium, occurred concurrently with the onset of ME/CFS.

## Case presentation

A 33-year-old male patient was referred to our department due to a seven-month history of psoriasis exacerbation, first diagnosed at the age of 17. He first received topical therapies, and his symptoms subsided when he was 20. He had not undergone any treatment since then until he experienced an exacerbation at the age of 33. Alongside the exacerbation of psoriasis, he developed right groin pain. Persistent profound fatigue, post-exertional malaise, sleep disturbances, and orthostatic tachycardia also developed at the same time. He was diagnosed with ME/CFS at a hospital specializing in neurology and psychiatry and treated with 2 mg/day of oral prednisolone, a selective serotonin reuptake inhibitor, an orexin receptor antagonist, a beta blocker, and a coenzyme Q10. He also used a proton pump inhibitor to suppress the drug-induced stomach discomfort.

**Figure 1:**
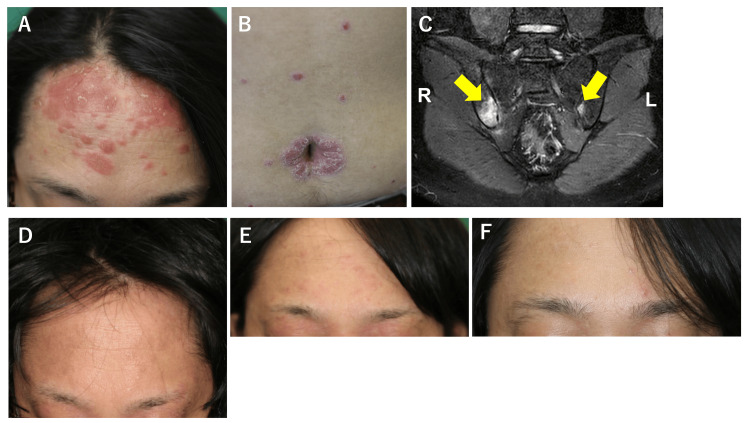
Clinical and radiological findings A, B) Scaly erythematous plaques on the forehead and the abdomen. C) Magnetic resonance imaging with short tau inversion recovery sequences revealing high signal intensity in both sacroiliac joints, with the right side showing more prominent findings (yellow arrows). D) Complete resolution of the skin lesion on the forehead after 12 weeks of treatment with ixekizumab. E) Eczematous lesions on the face after 22 weeks of treatment with ixekizumab. F) Improvement in eczematous lesions 4 weeks after switching to upadacitinib.

Initial examinations revealed scaly erythematous plaques scattered on the whole body, consistent with psoriasis (Fig. 1A, 1B). Onycholysis and splinter hemorrhages on fingernails were also observed, indicating the presence of nail psoriasis. The Psoriasis Area and Severity Index score was calculated as 17.7. The body mass index was 24.2 kg/m2, suggesting the absence of obesity. Routine laboratory tests were within normal limits. Magnetic resonance imaging using short tau inversion recovery sequences demonstrated high signal intensity in both sacroiliac joints, with more prominent findings on the right side (Fig. 1C). No significant abnormalities were detected in the lumbar spine. Treatment with ixekizumab was initiated, resulting in complete resolution of the skin lesions and significant improvement in joint pain after 12 weeks (Fig. 1D). However, at week 22 of ixekizumab therapy, eczematous lesions appeared predominantly on the face, leading to switching to upadacitinib (Fig. 1E), resulting in rapid improvement of the new lesions (Fig. 1F).

The patient has been receiving treatment with upadacitinib for two years without recurrence of psoriatic skin lesions and joint pain. Symptoms of ME/CFS slightly improved during the treatment compared to before treatment, but they have still remained.

## Discussion

Our case is interesting because of the simultaneous occurrence of psoriasis exacerbation, sacroiliitis development, and ME/CFS onset. The diagnostic criteria of ME/CFS were established by the Institute of Medicine, now called the National Academy of Medicine, in 2015 [3]. The following core symptoms are required for diagnosis: 1. A substantial reduction or impairment in the ability to engage in pre-illness levels of occupational, educational, social, or personal activities for more than six months, accompanied by profound fatigue that is not alleviated by rest. 2. Post-exertional malaise. 3. Unrefreshing sleep. 4. Cognitive impairment, such as brain fog, decreased concentration, and memory deficits, or orthostatic intolerance. In addition, the careful ruling out of other conditions such as hypothyroidism, depression, and autoimmune diseases is required.

Psoriatic arthritis (PsA) can induce some of the symptoms above, and there is a possibility that PsA mimicked ME/CFS in our case. However, the symptoms associated with profound fatigue remained despite the complete remission of skin and joint symptoms after the treatment with ixekizumab followed by upadacitinib, and the clinical diagnosis of ME/CFS was not changed, suggesting the presence of ME/CFS in our case. Although the pathogenesis of ME/CFS has not yet been fully elucidated, it is considered a systemic disorder arising from dysregulated interactions among the nervous, immune, and endocrine systems. Inflammatory cytokines may play a key role in this process. The largest study using serum of 186 ME/CFS patients revealed that the symptom severity significantly correlated with serum levels of several cytokines, such as IL-17F, CXCL1, and leptin [4]. The other group also showed the relationship between serum leptin levels and the severity of MF/CFS [5].

IL-17F, a member of the IL-17 cytokine family, acts on synovial cells and keratinocytes to induce the production of pro-inflammatory cytokines, as well as chemokines including CXCL1, thereby promoting both joint and skin inflammation and destruction. Serum IL-17F levels were increased in patients with psoriasis and PsA, and the levels were higher than those of IL-17A [6,7]. The polymorphism of the *IL17F *gene was strongly associated with increased psoriasis risk [8]. Leptin, an adipokine secreted by adipocytes, is also involved in the development of psoriasis by promoting Th1 and Th17 immune responses and suppressing regulatory T cells. Elevated serum leptin levels have been reported in patients with PsA [9]. Leptin mRNA expression levels in subcutaneous adipose tissues are correlated with the severity of psoriasis [10].

Collectively, MF/CFS and psoriasis share immunological abnormalities to some extent, and the activation of inflammatory cytokines and adipokines, such as IL-17F and leptin, may cause the concurrent exacerbation of psoriasis, development of sacroiliitis, and onset of ME/CFS in our case. However, as not only immunological abnormalities but also the nervous and endocrine system irregularity may be associated with ME/CFS, the cytokine-suppressing therapy may not induce the resolution of the disease, as shown in our case.

Eczematous lesions, which developed under the treatment of ixekizumab, may be caused by the augmentation of Th2 immune responses due to the suppression of Th17 immune responses by ixekizumab. There have been many reports on such paradoxical eczema caused by several biologics for psoriasis. A prospective cohort study from the United Kingdom and Ireland revealed that the adjusted incidence rates of paradoxical eczema were 1.22 per 100,000 person-years for IL-17 inhibitors, 0.94 per 100,000 person-years for tumor necrosis factor inhibitors, and 0.56 per 100,000 person-years for IL-23 inhibitors [11]. Similar to the previous report [12], the administration of upadacitinib, a Janus kinase 1 (JAK1) inhibitor, resulted in the remission of paradoxical eczema and the suppression of the recurrence of psoriasis in our case, probably because JAK1 inhibitors can suppress both Th2 and Th17 immune responses.

## Conclusions

ME/CFS, a systemic chronic disorder characterized by persistent and unexplained severe fatigue, can coincide with psoriasis. The coincidence may be caused by the shared immunological abnormalities of both diseases, but ME/CFS is associated not only with immunological abnormalities but also with nervous and endocrine system irregularities. The cytokine-suppressing therapy effective for psoriasis may not induce a complete resolution of ME/CFS, and personalized symptom management is required for the disease.
